# The shape of the radiation dose response for DNA double-strand break induction and repair

**DOI:** 10.1186/2041-9414-4-1

**Published:** 2013-03-22

**Authors:** Stephen Barnard, Simon Bouffler, Kai Rothkamm

**Affiliations:** 1 Health Protection Agency Centre for Radiation, Chemical and Environmental Hazards, Chilton, Didcot, Oxon OX11 0RQ, UK

**Keywords:** DNA double-strand break, Ionising radiation, Pulsed-field gel electrophoresis, Gamma-H2AX, Dose response, Low dose

## Abstract

DNA double-strand breaks are among the most deleterious lesions induced by ionising radiation. A range of inter-connected cellular response mechanisms has evolved to enable their efficient repair and thus protect the cell from the harmful consequences of un- or mis-repaired breaks which may include early effects such as cell killing and associated acute toxicities and late effects such as cancer. A number of studies suggest that the induction and repair of double-strand breaks may not always occur linearly with ionising radiation dose. Here we have aimed to identify and discuss some of the biological and methodological factors that can potentially modify the shape of the dose response curve obtained for these endpoints using the most common assays for double-strand breaks, pulsed-field gel electrophoresis and microscopic scoring of radiation-induced foci.

## Introduction

The topic of radiation risk to health, particularly at low-doses, i.e. a few to a few tens of millisievert or milligray in the case of sparsely ionising radiation, remains important, owing largely to the increasing (yet ever more effective) use of radiation in medical diagnosis, interventional radiology and also the treatment of cancers. For many years, our knowledge of both the health effects of and the molecular and cellular responses to ionising radiation exposure has been limited to the high dose range, above 100 mSv, due to a lack of sufficiently large and well controlled cohorts for epidemiological studies on one hand, and a lack of experimental tools for assessing low dose responses on the other hand. Based on the available evidence, a linear no-threshold model was generally assumed for cancer risk [1,2]. Over the past decade, however, considerable progress has been made, as illustrated by recent studies such as those on cancer risk associated with paediatric computed tomography (CT) scanning [3] and natural background radiation [4] which help reduce uncertainties about the shape of the dose response curve for cancer at low doses. In addition to cancer, cardiovascular disease has recently been identified as a potentially equally important contributor to radiation mortality [5]. Large funding programmes dedicated to experimental research into low dose effects and underlying mechanisms, such as the U.S. Department of Energy Low Dose program (http://lowdose.energy.gov/) and the previous (RISK-RAD, NOTE) and current EU initiatives DOREMI and MELODI (http://www.melodi-online.eu/) have supported the introduction of sensitive assays and biomarkers which provide new insights into the cellular and molecular responses at low doses [6].

Chromosomal DNA is the most important cellular target damaged by exposure to ionising radiation. Radiation-induced DNA lesions include abasic sites, oxidated bases and sugars, strand breaks and cross-links within or between the complementary DNA strands or between DNA and surrounding proteins. Importantly, radiation causes clusters of such lesions along the track of the ionising particle [7,8]. It is this ability to produce ‘locally multiply damaged sites’ containing two or more lesions within 1–2 helical turns of DNA [9], which distinguishes ionising radiation from the numerous other genotoxic agents that we encounter in our daily lives. One important clustered lesion is the DNA double-strand break (DSB). As it affects both complementary DNA strands, it is much harder to repair than any single-stranded lesions which can utilise the complementary sequence on the opposite strand as a template to ensure correct and efficient repair. In the following sections we highlight recent findings that may have a bearing on the shape of the dose response for DSB induction, signalling and repair and review methodological limitations. We concentrate on quantitative questions – numerous recent reviews have addressed the biochemical aspects of DNA damage signalling and repair (e.g. [10-13]) which are therefore not covered here. Also, the focus of this article is mainly on sparsely ionising radiations such as X- or gamma-rays; see [14] for a recent review of the DNA damage response to densely ionising radiation.

### DNA double-strand break induction by ionising radiation

Strand breaks are among the most highly studied DNA lesions induced by ionising radiation. This is partly because of their important contribution to the toxic, mutagenic, clastogenic and carcinogenic effects of radiation, but may also be explained by the availability of a wide range of detection and quantification methods for these particular lesions.

Ionising radiation-induced DNA strand breaks form following attack of the sugar phosphate backbone either by direct DNA radical production or by radicals formed through water radiolysis in the vicinity of the DNA (indirect effect). DSB form when two such nicks are present in opposite DNA strands within one or two helical turns. They seem to result mainly from the attack of multiple radical hits rather than the transfer of one radical between strands [15]. However, recent electron paramagnetic resonance spectroscopy results suggest that most DSB may not be derived from trappable radical pairs [16]. In contrast to DSB induction by H_2_O_2_ which shows a strong quadratic response at high concentrations [17] due to the interaction of independently produced radicals in DSB induction, radiation induces DSB by radicals originating from the same radiation track, and therefore linearly with dose, at least for doses up to several hundred gray [18].

The ratio of SSB to DSB yields produced by sparsely ionising radiation is commonly estimated to be on the order of 25–40, based on the detection of relaxed circular vs. linear plasmid DNA in agarose gels following irradiation of supercoiled circular plasmids. A recent study, however, which has utilised a direct end-labelling approach for SSB detection, suggests that the true SSB yield may be 10 fold higher than previously assumed [19]. The inability of the conventional plasmid agarose gel assay to detect additional strand breaks in the presence of one break (which should occur frequently, given the clustered distribution of ionisation events for radiation) may explain the lower yields reported in previous studies. Once confirmed, this finding may have implications for the relative risk attributed to radiation-induced SSB and DSB.

Up until about a decade ago it was impossible to study DSB induction at radiation doses of relevance in occupational or radiodiagnostic settings, due to the very limited sensitivity of the DSB detection methods available, such as neutral filter elution or pulsed-field gel electrophoresis (PFGE), which all measure DSB indirectly through the associated decrease in average molecular weight or length of chromosomal DNA and in general require doses of at least several gray to detect any significant effect, though a detection limit of less than 1 Gy was reported by one group for an optimised PFGE-based assay [20].

Over the past 15 years, radiation-induced foci (RIF), each representing hundreds to thousands of individual proteins involved in the DNA damage response which accumulate in the vicinity of a DSB [21,22], have been established as surrogate markers for DSB [23]. The most widely utilised markers include 1) the phosphorylated histone variant gamma-H2AX [24-26], 2) the autophosphorylated DNA damage kinase ATM-pS1981 [27] and 3) the mediator protein 53BP1 which may play an important role in chromatin remodelling at the break site [28]. Immunofluorescence microscopy enables the spatial localisation and quantification – manually or by image analysis software – of individual RIF, each thought to represent one or more DSB. More recently, live cell imaging of cells expressing fluorescent fusion proteins that are recruited to the sites of DSB has enabled detailed studies of the spatio-temporal dynamics of RIF [29,30].

### Electrophoretic DNA double-strand break assays

**Figure 1:**
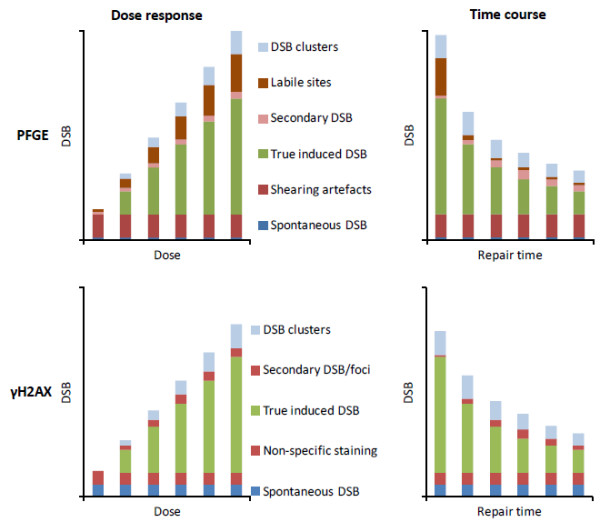
**Schematic dose response and time course for different classes of signals that contribute to DSB measurements. ***Top*: pulsed-field gel electrophoresis (PFGE). *Bottom*: fluorescence microscopic gamma-H2AX foci scoring. Note that the graphs are for illustrative purposes only. The values shown should not be taken as representative of the ‘typical’ contributions as they depend on numerous experimental factors.

Although initial DSB induction by ionising radiation can conceptually be expected to occur linearly with dose, there are number of factors that can affect DSB measurements by any of the above assays. Figure 1 illustrates some of the different classes of lesions that may affect dose response or time course relationships obtained with PFGE or foci assays for DSB induction and repair. Note that their relative contributions in individual studies may differ considerably from the values shown here, depending on a wide range of experimental parameters.

For PFGE-based assays, mechanical shearing and nuclease attack can contribute to DNA fragmentation that is independent of radiation exposure. This ‘noise’ limits the dose range available for investigation. Excessive DNA degradation can be minimised, but not eliminated, by embedding cells into low gelling temperature agarose, incubation of samples in chelating agents to inhibit nucleases prior to lysis and strict avoidance of any risk of contamination of samples with DNAse.

Measurement of the fraction of DNA released into the gel reveals a sigmoid dose response and requires accurate molecular weight analysis of fragment distributions and calibration with I-125 for absolute quantification of DSBs [31]. The use of rarely cutting restriction enzymes and probing of individual restriction fragments bypasses these complications and allows direct quantification of DSB yields in specific regions of the genome [32,33]. However, as both these PFGE approaches measure the electrophoretic migration of DNA fragments in the size range of hundreds to thousands of kilo base pairs (to achieve the highest assay sensitivity), they fail to detect clustered DSB which produce smaller fragments and therefore underestimate the total yield of DSB, especially for densely ionising radiations. Separate electrophoretic runs are required to resolve small and large fragments and thereby determine DSB yields more accurately, albeit only at very high doses [34].

**Figure 2:**
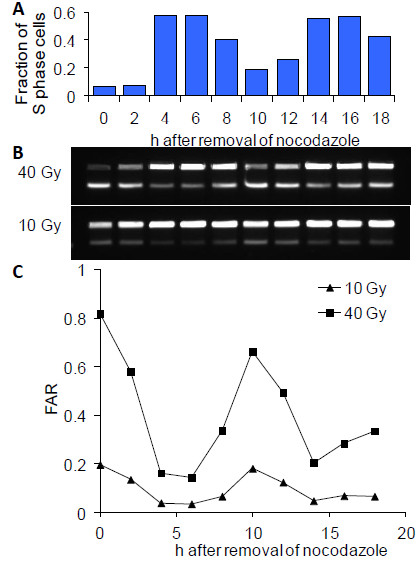
**The impact of S-phase DNA on pulsed-field electrophoretic DSB measurements.** Nocodazole-synchronised chicken DT40 pre-B cells were analysed 0–18 hours after removal of the drug. (**A**) Flow cytometric estimates of the S phase fraction. (**B**) Pulsed-field gel images of DNA migration following 40 and 10 Gy X-irradiation without repair incubation. (**C**): Fraction of DNA released (FAR) as a function of time after nocodazole removal. FAR values are inversely correlated to the fraction of S phase cells shown in the top diagram.

Replication forks can cause DNA molecules to be trapped in the agarose matrix, resulting in reduced mobility and associated underestimation of DNA breakage. Caution should therefore be used when interpreting PFGE results obtained with proliferating cells containing a significant S phase fraction (Figure 2; [35]).

In addition to inducing prompt DNA strand breaks, ionising radiation also induces heat- or alkaline-labile sites that are repaired by non-DSB pathways in the cell but can be converted into DSB during cell lysis and may contribute ~30% of all DSB measured using PFGE. Optimised ‘cold’ lysis and electrophoresis protocols have been established to eliminate these artefacts [36].

The high doses commonly used for PFGE studies may induce cell death in some cell types, potentially causing secondary DSB induction that may increase with repair time, and subsequent cell loss. Treatment with caspase inhibitors may help identify and control confounding apoptotic effects [37].

### Foci-based assays for DNA double-strand break analysis

A similar set of factors can also modulate DSB yields determined with RIF-based assays (Figure 1). Spontaneous DSB/foci levels have been observed to be much lower for non-cycling cells such as quiescent lymphocytes or tissues with a low turnover than for rapidly dividing cells and tissues. This effect is assumed to reflect replication-associated DNA breakage, with DSBs being carried over into subsequent cell cycle stages [38].

Artifactual foci formation in the absence of a DSB can be caused by non-specific staining or aggregate formation of the primary or secondary antibody. Gamma-H2AX antibodies may bind to and form foci at parts of the endoplasmic reticulum and/or Golgi vesicles (Scherthan, personal communication). Careful optimisation of staining conditions and close monitoring of the antibody performance are required in order to obtain consistent results. Staining artefacts can typically be distinguished from ‘true’ foci based on their different morphology. Subtle differences can, however, be lost in maximum projection images, so ‘live’ imaging of slides may be preferable.

Absolute yields of DSB per unit radiation dose change linearly with the DNA content, as observed for foci frequencies in cell lines with different DNA content [39]. Also, cells in late S/G2 were shown to form almost twice as many foci compared to G1 when exposed to the same radiation dose [38,40], though foci morphology and signal to noise ratios differ in different cell cycle phases, complicating such a comparison.

**Figure 3:**
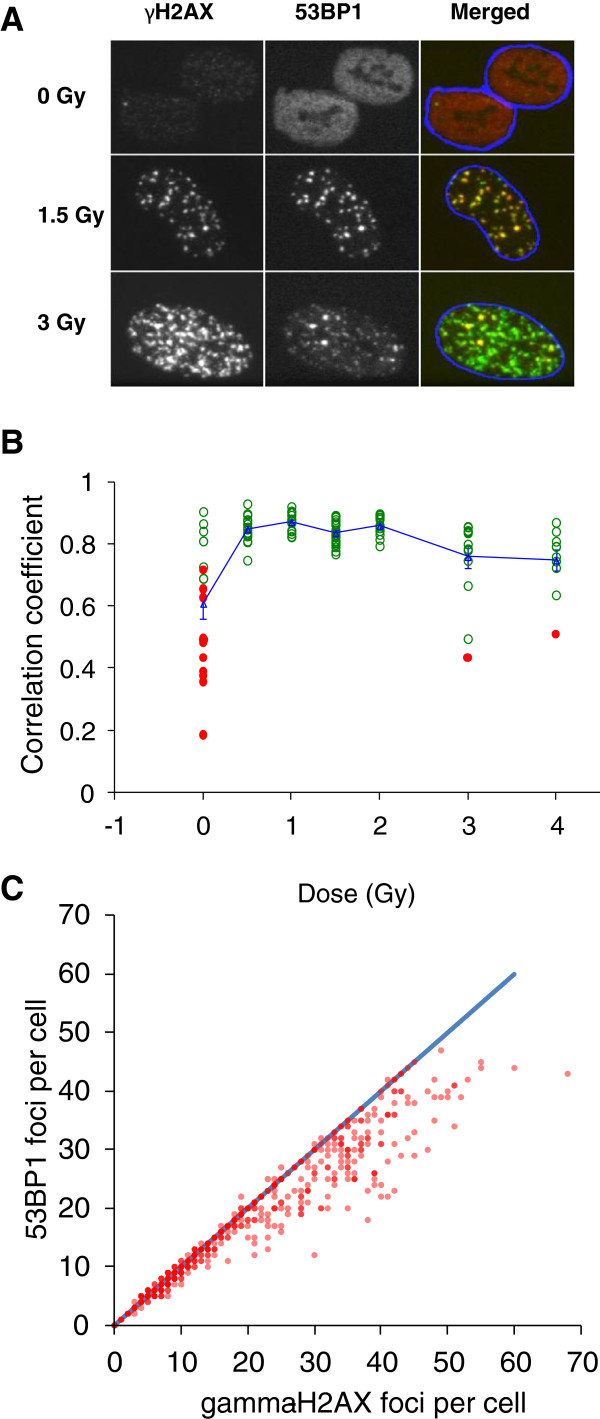
**gamma-H2AX and 53BP1 foci induction by X-rays.** (**A**) Immunofluorescence microscopy images were taken at 0.5h following X-irradiation of normal human fibroblasts. Each image is 20 μm wide. In the merged images 53BP1 is red, gamma-H2AX green and the nuclear margins are shown in blue. Co-localising foci appear yellow or orange. (**B**) Colocalisation analysis of gamma-H2AX and 53BP1 foci. Pearson’s correlation coefficients were calculated as described in [43]. A value of one represents total co-localisation. The significance of correlation coefficients was determined for individual cells using Costes’ spatial statistics approach [44]. Each point represents one cell. Filled red circles: non-significant, open green circles: significant correlation. Blue triangles, connected by blue line: mean correlation coefficient; error bars are standard errors from the analysis of 10–20 cells for each dose. (**C**) Gamma-H2AX versus 53BP1 foci count per cell, manually scored in the same 1,000 double-immunostained cells following exposure to a range of X-ray doses. Each data point corresponds to one cell. Shading of data point symbols reflects the number of coinciding points. The blue line indicates a 1:1 ratio.

Scoring of foci relies on setting threshold criteria for foci size, signal intensity and overall morphology of spots to distinguish ‘true’ foci from gamma-H2AX ‘speckles’ (which may form at the sites of transcription ‘bubbles’), antibody aggregates and non-specific binding of the antibody to other targets. Co-localisation of gamma-H2AX foci with 53BP1 is generally assumed to reflect true DSB [41,42] and double immunostaining for two foci-forming markers can therefore be used to validate the signal (Figure 3). Still, it has to be noted that very high spontaneous levels of apparently ‘real’ foci do seem to occur occasionally and it is not currently clear whether these really always reflect the DSB [21].

As foci form as a biological response to DNA damage, a period of at least several minutes post exposure is required before they can be microscopically detected; the exact minimum delay depends on the individual signal to noise level which varies between cell types and is also affected by experimental protocols and reagents used for foci immunostaining [24]. Consequently, some radiation-induced DSB may already have been repaired before the ‘initial’ foci yield can be determined. Similarly to PFGE, RIF assays fail to detect clustered DSB, as multiple DSB within <~0.5 μm would only be scored as one focus. In addition to DSB clustering caused by clustered ionisation events along the particle track, movement of multiple break ends (as far apart as 1–2 μm) into ‘repair centres’ [29] may introduce a second layer of clustering facilitated by an active biological response after physical damage induction. The latter effect may result in lower foci yields (per unit dose) at high compared to low doses. These effects could therefore contribute to an underestimation of DSB yields, based on foci counts, and to deviation from dose linearity.

Secondary gamma-H2AX foci have been reported in irradiated and bystander cells in association with replication stallage [45-47] and transcriptional activity [48]. Different types of secondary gamma-H2AX signals in UV-irradiated cells were recently reviewed [49], including weak and strong pan-nuclear gamma-H2AX induction in association with nucleotide excision repair and S phase apoptosis, respectively. Early apoptotic DNA breakage can also give rise to foci patterns that may sometimes be scored as residual radiation-induced foci, despite their distinct morphology [50]. As already mentioned for PFGE studies, apoptotic DSB can be identified and controlled using caspase inhibitors when analysing foci kinetics.

Current automated and manual foci scoring methods tend to underestimate RIF yields at high damage levels, thereby causing a deviation from the linear dose response relationship towards saturation. Using the highest possible optical resolution, manual, rather than automated scoring and scoring of gamma-H2AX rather than 53BP1 foci at high damage levels (see Figure 3; also discussed in [41]) may help minimise this effect.

Notwithstanding all the potential technical caveats in measuring radiation-induced DSB, and the complex biological processes that may result in secondary DSB formation, as highlighted in the above paragraphs, DSB appear to be induced linearly with radiation dose for a wide range of radiation types and doses. At low doses, however, the situation is less clear. Specifically, supralinear foci induction in lymphocytes from paediatric patients was reported recently for both *in vivo* and ex vivo exposures to diagnostic X-ray doses [51]. As this effect was more pronounced when whole blood was irradiated rather than isolated lymphocytes [52], bystander-type effects were suggested as a possible explanation. However, Beels and colleagues observed this effect only for low dose X- but not gamma-rays. The clustering of adjacent foci into repair centres, as reported in [29,52], may offer an alternative explanation. Overall, there is still controversy over the shape of the dose response for foci induction at low doses, due to a diminishing signal to noise ratio, lack of sensitive assays to confirm RIF data and the need for larger data sets to obtain conclusive results at doses in the milligray range.

### Repair of radiation-induced DSB

PFGE studies suggest fairly dose-independent kinetics of DSB rejoining which follow a biexponential decay, allowing repair half-times for a fast (of the order of 10–30 minutes) and a slow component (a few hours) to be calculated [53]. The biphasic nature of DSB repair kinetics has been associated with different repair pathways [53], the complexity of break ends [54,55] or surrounding chromatin structures [56], requiring additional processing or remodelling steps, respectively. It should be noted that most PFGE data were obtained using tens of gray of sparsely ionising radiation, and that pre-electrophoretic cell lysis likely converted radiation-induced heat/alkaline-labile sites into DSB, resulting in a larger fast component [36]. As repair of these labile sites does not seem to require functional end-joining, XRCC1 or poly(ADP-ribose) polymerase 1 [36,57], their biological significance remains unclear.

Dose-independent, biexponential kinetics are also widely observed using foci assays, but typically with longer half times; e.g. Horn et al. [41] reported 1.5 h and 1.5 days for the fast and slow component, respectively, following 0.5 – 4 Gy X-irradiation of human lymphocytes. Apart from the issue with heat/alkaline labile sites mentioned above, a number of other factors may contribute to the delayed foci loss compared to PFGE-based DSB rejoining kinetics: 1) The formation of foci over several minutes following irradiation means that some rapidly repaired DSB may never be registered as foci. 2) Whilst a few publications have reported maximum foci yields within the first few minutes post exposure, followed by rapid loss with kinetics compatible with those reported for PFGE [58-60], most studies observed foci counts peaking later, at 0.5-1h post exposure, and reaching a lower maximum yield per unit dose, followed by a slower loss. As discussed in [24], these discrepancies may be explained by differences in the detectibility of early, i.e. small, gamma-H2AX foci, depending on the signal-to-noise ratio of immunostained samples. The half times reported in [41] were obtained using 0.5 h post exposure as the earliest time point, thus missing out on a large part of the fast component measured in PFGE experiments. 3) Dose ranges available for DSB repair studies are one to three orders of magnitude lower for RIF compared to PFGE assays [58]. Consequently, a lack of induction of DSB repair or secondary DSB formation which may contribute significantly to overall DSB frequencies only at low doses, may result in apparently slower kinetics following low dose exposure, or in the long-term persistence of residual foci [58,61,62]. 4) It is not clear how closely RIF loss follows the resealing of DSB ends. Resolution of foci is facilitated by a number of protein phosphatases whose complex roles in the DNA damage response are not very well understood yet [63].

Neumaier et al. [29] reported slower foci loss at higher doses, in line with the hypothesis that multiple DSB may congregate into one shared ‘repair center’, represented by one gamma-H2AX focus, resulting in longer overall persistence of such a focus, until all breaks contained in it are repaired. This concept has some intriguing implications for the way chromosomal rearrangements arise. The crucial impact of spatial and temporal proximity of DSB on mis-rejoining of break ends had already been highlighted in previous experimental and modelling studies (reviewed in e.g. [64-70]). However, it was only more recent work using high resolution interphase in situ hybridization [71] and time lapse imaging of RIF [29] that has unveiled the considerable intermingling of chromosome territories and DSB, respectively, thus explaining the large interaction distances for DSB of 1–2 μm that had been estimated in the earlier modelling studies of the quadratic dose dependence of chromosome rearrangement formation at high doses of sparsely ionising radiation.

## Conclusions

In conclusion, the seemingly simple task to determine the shape of the dose response curve for DSB induction and repair is, at closer inspection, associated with numerous technical and conceptual caveats and uncertainties that should be considered when interpreting any experimental data. New assays have considerably advanced our understanding of the way cells respond to radiation-induced damage. It is becoming increasingly clear that multiple biological processes, but also methodological factors, may cause the dose response to deviate from linearity. Estimating their impact on the effect of radiation at a tissue or organism level remains a major challenge. In this context extrapolation of results from single experimental studies to draw conclusions on the most appropriate dose–response model to use for the protection of populations against the health effects of ionising radiation may be seen as unwise and potentially misleading.

## Competing interests

The authors declare that they have no competing interests.

## Authors’ contributions

SB and KR carried out the initial literature review and drafted the manuscript. SDB contributed to the design and helped to draft the manuscript. All authors read and approved the final manuscript.

## References

[B1] Mullenders L Atkinson M Paretzke H Sabatier L Bouffler S Assessing cancer risks of low-dose radiation Nat Rev Cancer 2009 9 596 604 10.1038/nrc2677 19629073

[B2] Little MP Wakeford R Tawn EJ Bouffler SD Berrington de Gonzalez A Risks associated with low doses and low dose rates of ionizing radiation: why linearity may be (almost) the best we can do Radiology 2009 251 6 12 10.1148/radiol.2511081686 19332841 PMC2663578

[B3] Pearce MS Salotti JA Little MP McHugh K Lee C Kim KP Howe NL Ronckers CM Rajaraman P Sir Craft AW Parker L Berrington de Gonzalez A Radiation exposure from CT scans in childhood and subsequent risk of leukaemia and brain tumours: a retrospective cohort study Lancet 2012 380 499 505 10.1016/S0140-6736(12)60815-0 22681860 PMC3418594

[B4] Kendall GM Little MP Wakeford R Bunch KJ Miles JCH Vincent TJ Meara JR Murphy MFG A record-based case–control study of natural background radiation and the incidence of childhood leukaemia and other cancers in Great Britain during 1980–2006 Leukemia 2013 27 3 9 10.1038/leu.2012.151 22766784 PMC3998763

[B5] Little MP Azizova TV Bazyka D Bouffler SD Cardis E Chekin S Chumak VV Cucinotta FA de Vathaire F Hall P Harrison JD Hildebrandt G Ivanov V Kashcheev VV Klymenko SV Kreuzer M Laurent O Ozasa K Schneider T Tapio S Taylor AM Tzoulaki I Vandoolaeghe WL Wakeford R Zablotska LB Zhang W Lipshultz SE Systematic Review and Meta-analysis of Circulatory Disease from Exposure to Low-Level Ionizing Radiation and Estimates of Potential Population Mortality Risks Environ Health Perspect 2012 120 1503 1511 10.1289/ehp.1204982 22728254 PMC3556625

[B6] Pernot E Hall J Baatout S Benotmane MA Blanchardon E Bouffler S El Saghire H Gomolka M Guertler A Harms-Ringdahl M Jeggo P Kreuzer M Laurier D Lindholm C Mkacher R Quintens R Rothkamm K Sabatier L Tapio S de Vathaire F Cardis E Ionizing radiation biomarkers for potential use in epidemiological studies Mutat Res 2012 751 258 286 10.1016/j.mrrev.2012.05.003 22677531

[B7] Cadet J Ravanat J Tavernaporro M Menoni H Angelov D Oxidatively generated complex DNA damage: Tandem and clustered lesions Cancer Lett 2012 327 5 15 10.1016/j.canlet.2012.04.005 22542631

[B8] Eccles LJ O’Neill P Lomax ME Delayed repair of radiation induced clustered DNA damage: friend or foe? Mutat Res 2011 711 134 141 10.1016/j.mrfmmm.2010.11.003 21130102 PMC3112496

[B9] Ward JF DNA damage produced by ionizing radiation in mammalian cells: identities, mechanisms of formation, and reparability Prog Nucleic Acid Res Mol Biol 1988 35 95 125 3065826 10.1016/s0079-6603(08)60611-x

[B10] Thompson LH Recognition, signaling, and repair of DNA double-strand breaks produced by ionizing radiation in mammalian cells: the molecular choreography Mutat Res 2012 751 158 246 10.1016/j.mrrev.2012.06.002 22743550

[B11] Ciccia A Elledge SJ The DNA damage response: making it safe to play with knives Mol Cell 2010 40 179 204 10.1016/j.molcel.2010.09.019 20965415 PMC2988877

[B12] Polo SE Jackson SP Dynamics of DNA damage response proteins at DNA breaks: a focus on protein modifications Genes Dev 2011 25 409 433 10.1101/gad.2021311 21363960 PMC3049283

[B13] Giglia-Mari G Zotter A Vermeulen W DNA damage response Cold Spring Harb Perspect Biol 2011 3 a000745 10.1101/cshperspect.a000745 20980439 PMC3003462

[B14] Asaithamby A Chen DJ Mechanism of cluster DNA damage repair in response to high-atomic number and energy particles radiation Mutat Res 2011 711 87 99 10.1016/j.mrfmmm.2010.11.002 21126526 PMC3318975

[B15] Milligan JR Ng JY Wu CC Aguilera JA Fahey RC Ward JF DNA repair by thiols in air shows two radicals make a double-strand break Radiat Res 1995 143 273 280 10.2307/3579213 7652164

[B16] Peoples AR Mercer KR Bernhard WA What fraction of DNA double-strand breaks produced by the direct effect is accounted for by radical pairs? J Phys Chem B 2010 114 9283 9288 10.1021/jp103362z 20583765 PMC2914509

[B17] Dahm-Daphi J Sass C Alberti W Comparison of biological effects of DNA damage induced by ionizing radiation and hydrogen peroxide in CHO cells Int J Radiat Biol 2000 76 67 75 10.1080/095530000139023 10665959

[B18] Löbrich M Rydberg B Cooper P Repair of x-ray-induced DNA double-strand breaks in specific Not I restriction fragments in human fibroblasts: joining of correct and incorrect ends Proc Natl Acad Sci USA 1995 92 12050 12054 10.1073/pnas.92.26.12050 8618842 PMC40294

[B19] Balagurumoorthy P Adelstein SJ Kassis AI Novel method for quantifying radiation-induced single-strand-break yields in plasmid DNA highlights 10-fold discrepancy Anal Biochem 2011 417 242 246 10.1016/j.ab.2011.06.023 21741945 PMC3184882

[B20] Gradzka I Iwanenko T A non-radioactive, PFGE-based assay for low levels of DNA double-strand breaks in mammalian cells DNA Repair (Amst) 2005 4 1129 1139 10.1016/j.dnarep.2005.06.001 15994132

[B21] Costes SV Chiolo I Pluth JM Barcellos-Hoff MH Jakob B Spatiotemporal characterization of ionizing radiation induced DNA damage foci and their relation to chromatin organization Mutat Res 2010 704 78 87 10.1016/j.mrrev.2009.12.006 20060491 PMC3951968

[B22] Bekker-Jensen S Mailand N Assembly and function of DNA double-strand break repair foci in mammalian cells DNA Repair 2010 9 1219 1228 10.1016/j.dnarep.2010.09.010 21035408

[B23] Feuerhahn S Egly J Tools to study DNA repair: what’s in the box? Trends Genet 2008 24 467 474 10.1016/j.tig.2008.07.003 18675488

[B24] Rothkamm K Horn S Gamma-H2AX as protein biomarker for radiation exposure Ann Ist Super Sanita 2009 45 265 271 19861731

[B25] Rogakou EP Pilch DR Orr AH Ivanova VS Bonner WM DNA double-stranded breaks induce histone H2AX phosphorylation on serine 139 J Biol Chem 1998 273 5858 5868 10.1074/jbc.273.10.5858 9488723

[B26] Mah L Orlowski C Ververis K El-Osta A Karagiannis TC Utility of gammaH2AX as a molecular marker of DNA double-strand breaks in nuclear medicine: applications to radionuclide therapy employing auger electron-emitting isotopes Curr Radiopharm 2011 4 59 67 10.2174/1874471011104010059 22191615

[B27] So S Davis AJ Chen DJ Autophosphorylation at serine 1981 stabilizes ATM at DNA damage sites J Cell Biol 2009 187 977 990 10.1083/jcb.200906064 20026654 PMC2806275

[B28] Noon AT Goodarzi AA 53BP1-mediated DNA double strand break repair: insert bad pun here DNA Repair 2011 10 1071 1076 10.1016/j.dnarep.2011.07.012 21868291

[B29] Neumaier T Swenson J Pham C Polyzos A Lo AT Yang P Dyball J Asaithamby A Chen DJ Bissell MJ Thalhammer S Costes SV Evidence for formation of DNA repair centers and dose–response nonlinearity in human cells Proc Natl Acad Sci USA 2012 109 443 448 10.1073/pnas.1117849108 22184222 PMC3258602

[B30] Asaithamby A Chen DJ Cellular responses to DNA double-strand breaks after low-dose gamma-irradiation Nucleic Acids Res 2009 37 3912 3923 10.1093/nar/gkp237 19401436 PMC2709554

[B31] Ager DD Dewey WC Calibration of pulsed field gel electrophoresis for measurement of DNA double-strand breaks Int J Radiat Biol 1990 58 249 259 10.1080/09553009014551601 1974573

[B32] Rothkamm K Löbrich M Misrejoining of DNA double-strand breaks in primary and transformed human and rodent cells: a comparison between the HPRT region and other genomic locations Mutat Res 1999 433 193 205 10.1016/S0921-8777(99)00008-7 10343652

[B33] Löbrich M Ikpeme S Kiefer J DNA double-strand break measurement in mammalian cells by pulsed-field gel electrophoresis: an approach using restriction enzymes and gene probing Int J Radiat Biol 1994 65 623 630 10.1080/09553009414550731 7912711

[B34] Löbrich M Cooper P Rydberg B Non-random distribution of DNA double-strand breaks induced by particle irradiation Int J Radiat Biol 1996 70 493 503 10.1080/095530096144680 8947529

[B35] Mateos S Gordon AT Steel GG McMillan TJ Cell-cycle variation in DNA migration in pulsed-field gel electrophoresis Int J Radiat Biol 1996 69 687 693 10.1080/095530096145427 8691020

[B36] Stenerlöw B Karlsson KH Cooper B Rydberg B Measurement of prompt DNA double-strand breaks in mammalian cells without including heat-labile sites: results for cells deficient in nonhomologous end joining Radiat Res 2003 159 502 510 10.1667/0033-7587(2003)159[0502:MOPDDS]2.0.CO;2 12643795

[B37] Balart J Pueyo G de Llobet LI Baro M Sole X Marin S Casanovas O Mesia R Capella G The use of caspase inhibitors in pulsed-field gel electrophoresis may improve the estimation of radiation-induced DNA repair and apoptosis Radiat Oncol 2011 6 6 10.1186/1748-717X-6-6 21235815 PMC3025872

[B38] Rothkamm K Krüger I Thompson LH Löbrich M Pathways of DNA double-strand break repair during the mammalian cell cycle Mol Cell Biol 2003 23 5706 5715 10.1128/MCB.23.16.5706-5715.2003 12897142 PMC166351

[B39] Wardman P Rothkamm K Folkes LK Woodcock M Johnston PJ Radiosensitization by nitric oxide at low radiation doses Radiat Res 2007 167 475 484 10.1667/RR0827.1 17388699

[B40] Bauerschmidt C Arrichiello C Burdak-Rothkamm S Woodcock M Hill MA Stevens DL Rothkamm K Cohesin promotes the repair of ionizing radiation-induced DNA double-strand breaks in replicated chromatin Nucleic Acids Res 2010 38 477 487 10.1093/nar/gkp976 19906707 PMC2811025

[B41] Horn S Barnard S Rothkamm K Gamma-H2AX-based dose estimation for whole and partial body radiation exposure PLoS One 2011 6 e25113 10.1371/journal.pone.0025113 21966430 PMC3179476

[B42] de Feraudy S Revet I Bezrookove V Feeney L Cleaver JE A minority of foci or pan-nuclear apoptotic staining of gammaH2AX in the S phase after UV damage contain DNA double-strand breaks Proc Natl Acad Sci USA 2010 107 6870 6875 10.1073/pnas.1002175107 20351298 PMC2872460

[B43] Manders EM Stap J Brakenhoff GJ van Driel R Aten JA Dynamics of three-dimensional replication patterns during the S-phase, analysed by double labelling of DNA and confocal microscopy J Cell Sci 1992 103 857 862 1478975 10.1242/jcs.103.3.857

[B44] Costes SV Daelemans D Cho EH Dobbin Z Pavlakis G Lockett S Automatic and quantitative measurement of protein-protein colocalization in live cells Biophys J 2004 86 3993 4003 10.1529/biophysj.103.038422 15189895 PMC1304300

[B45] Sedelnikova OA Nakamura A Kovalchuk O Koturbash I Mitchell SA Marino SA Brenner DJ Bonner WM DNA double-strand breaks form in bystander cells after microbeam irradiation of three-dimensional human tissue models Cancer Res 2007 67 4295 4302 10.1158/0008-5472.CAN-06-4442 17483342

[B46] Groth P Orta ML Elvers I Majumder MM Lagerqvist A Helleday T Homologous recombination repairs secondary replication induced DNA double-strand breaks after ionizing radiation Nucleic Acids Res 2012 40 6585 6594 10.1093/nar/gks315 22505579 PMC3413124

[B47] Burdak-Rothkamm S Short SC Folkard M Rothkamm K Prise KM ATR-dependent radiation-induced gamma H2AX foci in bystander primary human astrocytes and glioma cells Oncogene 2007 26 993 1002 10.1038/sj.onc.1209863 16909103

[B48] Dickey JS Baird BJ Redon CE Avdoshina V Palchik G Wu J Kondratyev A Bonner WM Martin OA Susceptibility to bystander DNA damage is influenced by replication and transcriptional activity Nucleic Acids Res 2012 40 10274 10286 10.1093/nar/gks795 22941641 PMC3488239

[B49] Cleaver JE gammaH2Ax: biomarker of damage or functional participant in DNA repair “all that glitters is not gold!” Photochem Photobiol 2011 87 1230 1239 10.1111/j.1751-1097.2011.00995.x 21883247

[B50] Rogakou EP Nieves-Neira W Boon C Pommier Y Bonner WM Initiation of DNA fragmentation during apoptosis induces phosphorylation of H2AX histone at serine 139 J Biol Chem 2000 275 9390 9395 10.1074/jbc.275.13.9390 10734083

[B51] Beels L Bacher K De Wolf D Werbrouck J Thierens H Gamma-H2AX foci as a biomarker for patient X-ray exposure in pediatric cardiac catheterization: are we underestimating radiation risks? Circulation 2009 120 1903 1909 10.1161/CIRCULATIONAHA.109.880385 19858412

[B52] Beels L Werbrouck J Thierens H Dose response and repair kinetics of gamma-H2AX foci induced by in vitro irradiation of whole blood and T-lymphocytes with X- and gamma-radiation Int J Radiat Biol 2010 86 760 768 10.3109/09553002.2010.484479 20597840

[B53] Iliakis G Wang H Perrault AR Boecker W Rosidi B Windhofer F Wu W Guan J Terzoudi G Pantelias G Mechanisms of DNA double strand break repair and chromosome aberration formation Cytogenet Genome Res 2004 104 14 20 10.1159/000077461 15162010

[B54] Strande NT Waters CA Ramsden DA Resolution of complex ends by Nonhomologous end joining - better to be lucky than good? Genome Integr 2012 3 10 10.1186/2041-9414-3-10 23276302 PMC3547747

[B55] Cucinotta FA Pluth JM Anderson JA Harper JV O’Neill P Biochemical kinetics model of DSB repair and induction of gamma-H2AX foci by non-homologous end joining Radiat Res 2008 169 214 222 10.1667/RR1035.1 18220463

[B56] Goodarzi AA Jeggo P Löbrich M The influence of heterochromatin on DNA double strand break repair: Getting the strong, silent type to relax DNA Repair 2010 9 1273 1282 10.1016/j.dnarep.2010.09.013 21036673

[B57] Karlsson KH Radulescu I Rydberg B Stenerlow B Repair of radiation-induced heat-labile sites is independent of DNA-PKcs, XRCC1 and PARP Radiat Res 2008 169 506 512 10.1667/RR1076.1 18439038

[B58] Rothkamm K Löbrich M Evidence for a lack of DNA double-strand break repair in human cells exposed to very low x-ray doses Proc Natl Acad Sci USA 2003 100 5057 5062 10.1073/pnas.0830918100 12679524 PMC154297

[B59] Rothkamm K Balroop S Shekhdar J Fernie P Goh V Leukocyte DNA damage after multi-detector row CT: a quantitative biomarker of low-level radiation exposure Radiology 2007 242 244 251 10.1148/radiol.2421060171 17185671

[B60] Rogakou E Boon C Redon C Bonner W Megabase chromatin domains involved in DNA double-strand breaks in vivo J Cell Biol 1999 146 905 916 10.1083/jcb.146.5.905 10477747 PMC2169482

[B61] Ojima M Furutani A Ban N Kai M Persistence of DNA double-strand breaks in normal human cells induced by radiation-induced bystander effect Radiat Res 2011 175 90 96 10.1667/RR2223.1 21175351

[B62] Grudzenski S Raths A Conrad S Rube CE Löbrich M Inducible response required for repair of low-dose radiation damage in human fibroblasts Proc Natl Acad Sci USA 2010 107 14205 14210 10.1073/pnas.1002213107 20660770 PMC2922519

[B63] Shimada M Nakanishi M Response to DNA damage: why do we need to focus on protein phosphatases? Front Oncol 2013 3 8 23386996 10.3389/fonc.2013.00008 PMC3560363

[B64] Savage JR Insight into sites Mutat Res 1996 366 81 95 10.1016/S0165-1110(96)90030-5 9001576

[B65] Savage JR Cancer. Proximity matters Science 2000 290 62 63 10.1126/science.290.5489.62 11183150

[B66] Savage JR Interchange and intra-nuclear architecture Environ Mol Mutagen 1993 22 234 244 10.1002/em.2850220410 8223504

[B67] Sachs RK Chen AM Brenner DJ Review: proximity effects in the production of chromosome aberrations by ionizing radiation Int J Radiat Biol 1997 71 1 19 10.1080/095530097144364 9020958

[B68] Rothkamm K Löbrich M Misrepair of radiation-induced DNA double-strand breaks and its relevance for tumorigenesis and cancer treatment Int J Oncol 2002 21 433 440 12118342

[B69] Kühne M Rothkamm K Löbrich M Physical and biological parameters affecting DNA double strand break misrejoining in mammalian cells Radiat Prot Dosimetry 2002 99 129 132 10.1093/oxfordjournals.rpd.a006742 12194264

[B70] Hlatky L Sachs RK Vazquez M Cornforth MN Radiation-induced chromosome aberrations: insights gained from biophysical modeling BioEssays 2002 24 714 723 10.1002/bies.10126 12210532

[B71] Branco MR Pombo A Intermingling of chromosome territories in interphase suggests role in translocations and transcription-dependent associations PLoS Biol 2006 4 e138 10.1371/journal.pbio.0040138 16623600 PMC1440941

